# The axillary vein and its tributaries are not in the mirror image of the axillary artery and its branches

**DOI:** 10.1371/journal.pone.0210464

**Published:** 2019-01-10

**Authors:** HyeYeon Lee, JongHo Bang, SooJung Kim, HeeJun Yang

**Affiliations:** 1 Department of Anatomy, Yonsei University College of Medicine, Seoul, Republic of Korea; 2 Department of Anatomy, Gachon University College of Medicine, Incheon, Republic of Korea; IRCCS Policlinico S.Donato, ITALY

## Abstract

**Introduction:**

The axillary and cephalic veins are used for various clinical purposes but their anatomy is not fully understood. Increased knowledge and information about them as well as superficial veins in the upper arm would be useful.

**Objective:**

The aim of this study is to contribute to the literature regarding the anatomy of the venous drainage of the upper extremity.

**Methods:**

The veins of forty upper extremities from twenty one adult cadavers were injected and their axillary regions dissected. The course and pattern of drainage of the venous tributaries in the axillary region were identified and recorded.

**Results:**

The basilic, brachial, subscapular, lateral thoracic and superior thoracic veins drained mainly into the axillary vein, in common with most textbook descriptions. However, the thoracoacromial veins were observed to drain into the cephalic vein in 70.0% of upper limbs. In addition, a venous channel connecting the distal part and proximal part of the axilla was found along the posterolateral wall of the axilla in 77.5% of the upper limbs. In 95.0% of upper limbs, we discovered a superficial vein which ran from the axillary base and drained directly into the axillary vein.

**Conclusion:**

The veins from the inferomedial part of the axilla drain into the axillary vein, whereas the veins from the superolateral part of the axilla drain into the cephalic vein. The venous drainage of the axilla is variable and in common with venous drainage elsewhere, does not necessarily follow the pattern of the arterial supply.

## Introduction

There is a growing need for knowledge of the anatomy of the veins in the upper limb that can be applied to clinical situations such as peripheral vascular access, peripherally inserted central catheter implantation, hemodialysis, salvage operation, and cardiac device implantation [1–5]. Although central venous catheters are used across the range of medical and surgical specialities [6], it is suggested that central venous access should be avoided if possible because of potentially life-threatening complications. These include pneumothorax, hemothorax, cardiac tamponade, pleural effusion and phrenic nerve paresis [7]. A well-documented correlation between central venous catheters and central vein stenosis affects up to half of patients with a history of central venous catheter placement [8]. For peripheral intravenous catheterization, upper limb veins are the site of choice, and preferred to lower limb veins due to their accessibility and the lower risk of blood stream infection [6, 9].

Not all veins in the upper limb, however, may be acceptable for catheterization. The selection of the appropriate vein for invasive procedures such as a cardiac device implantation is important to reduce the risk of vascular complications [10]. Long–term usage of a peripheral vein may also lead to venous damage, as the improvements in medical care have increased the quality of life and the life expectancy of patients [11].

Among the veins in the upper extremity, the cephalic and axillary veins are regarded as being suitable for venous access in the upper limb. The cephalic vein is widely used as the first choice for venous cutdown for the insertion of chronic indwelling central venous access devices and pacemaker and defibrillator lead insertion, with a reduced rate of acute complications and higher success rate [12–15].

The axillary vein, which is the continuation of the basilic vein at the lower border of teres major [16], is the preferred access site for cardiac pacing leads because it is large enough to accommodate multiple leads, whilst minimizing the associated risks of pneumothorax, mediastinal hematoma, hemothorax, and tracheal injury [5, 17]. In premature neonates, compared to other sites, axillary peripheral vascular access lines were less likely to have complications and more likely to endure compared with peripheral access lines at other insertion sites. In severely burned patients, approach to the central vein through the axillary vein is regarded as a reasonable alternative to approach through other superficial veins, due to its protected location between the torso and arm [18].

Despite the favored use of the cephalic and axillary veins for various clinical purposes, their anatomy is not fully understood. For example, the axillary vein has been thought to receive tributaries that parallel the branches of the axillary artery [19], however this is not consistent with our findings during cadaveric dissections. Such misconceptions could lead to venepuncture failure, venous obstruction or congestion after breast surgery, or flap failure [20–23].

In addition, identifying a safe alternative entry vessel is crucial for the treatment of patients when access via the usual insertion sites is impossible [24]. Increased knowledge and information about superficial veins in the upper arm would help determine alternate means of venous access in cases where planned venous access is inadequate [14]. The aim of the present study is to identify and record the anatomy of the cephalic and axillary veins and their tributaries.

## Materials and methods

Anatomical dissections were performed on 40 upper extremities from 21 embalmed adult Korean cadavers comprising 13 males and eight females (mean age, 73.3 years; range, 54–90 years). In two cadavers, the upper extremity in one side was abandoned, because the tissues were of an inappropriate condition for anatomical study.

For the injection of the blue-colored dye, the blood clots in the subclavian vein were washed out with tap water using 50 ml syringe. Microfil, a radiopaque dye kit (FlowTech, Carver, MA, USA) was used for the injection. The pigmented compound, diluents and curing agent were mixed at volume ratio of 50:50:4. The mixture was injected into the subclavian vein.

After the pectoralis major and pectoralis minor were cut from the anterior thoracic wall, the axillary sheath was removed and the axillary vein and its tributaries were identified. The lateral and medial brachial veins, which are the vena comitantes of the brachial artery, were identified. The basilic vein was identified in the subcutaneous tissue of arm.

The cephalic vein was identified in the subcutaneous tissue of the arm and shoulder. Any venous tributaries to the cephalic vein in the arm, axilla and shoulder were identified.

Next, any of the veins in the axillary region including the superior thoracic vein, lateral thoracic vein, thoracoacromial vein, anterior circumflex humeral vein, posterior circumflex humeral vein and subscapular vein, were identified and traced until they drained into a recipient vessel. In cases where a vein drained to multiple recipient vessels, the largest connection was considered as the main functional drainage of the vein.

In addition, any arterial variations associated with the variation in the venous drainage was to be identified.

To find out any difference in the drainage of the veins between left and right sides, Fisher’s exact test was performed on the destination of every vein in this study. Fisher’s exact test was conducted to find any differences in the drainage between the veins. The test was also conducted to find any difference between right upper limbs and left upper limbs. MedCalc version 18.10.2 (MedCalc, Mariakerke, Belgium) was used for statistical analysis in this study. Statistical significance was set at *p* < 0.05 for all tests.

This study was conducted under the direction by Surgical Training Center of Yonsei University Medical College, Seoul, Korea. The cadaveric materials were donated through willed body donation program of Yonsei University. The authors did not have access to the records of donor’s medical history. The Act of Corpse Dissection and Preservation of Korea was observed throughout the study.

## Results

### The course of the cephalic vein and its tributaries

The course and drainage of the cephalic vein in most upper limbs was identical to that described in textbooks, with the majority of them (97.5%), draining into the proximal third of the axillary vein. The only exception was that, in one upper limb in this study, the cephalic vein drained into the basilic vein below the axillary region.

**Fig 1:**
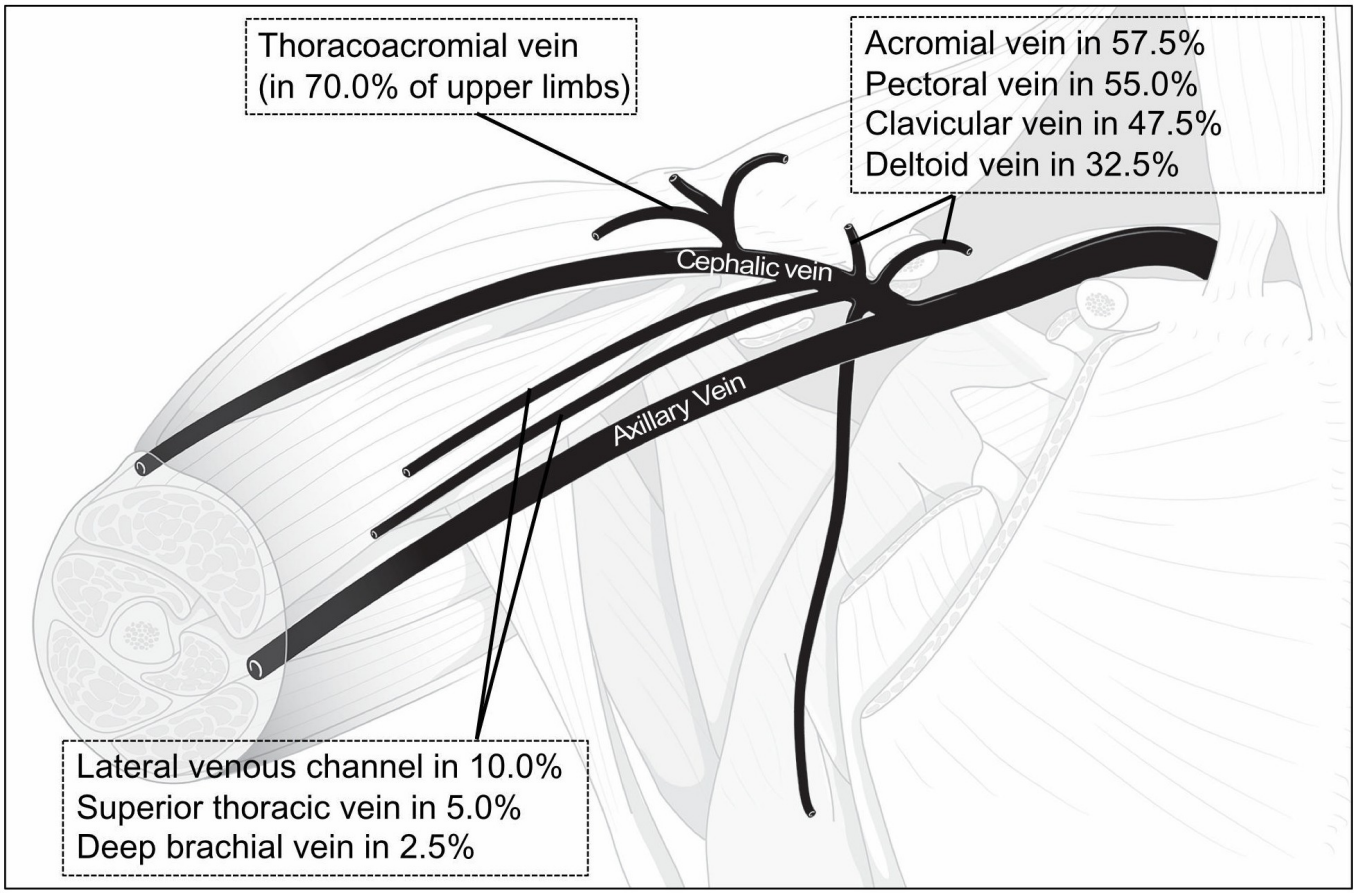
The frequencies / rates of the drainage of veins into the cephalic vein in the upper limbs. The tributaries are the thoracoacromial vein, acromial vein, pectoral vein, clavicular vein, deltoid vein, lateral venous channel, superior thoracic vein and deep brachial vein.

**Fig 2:**
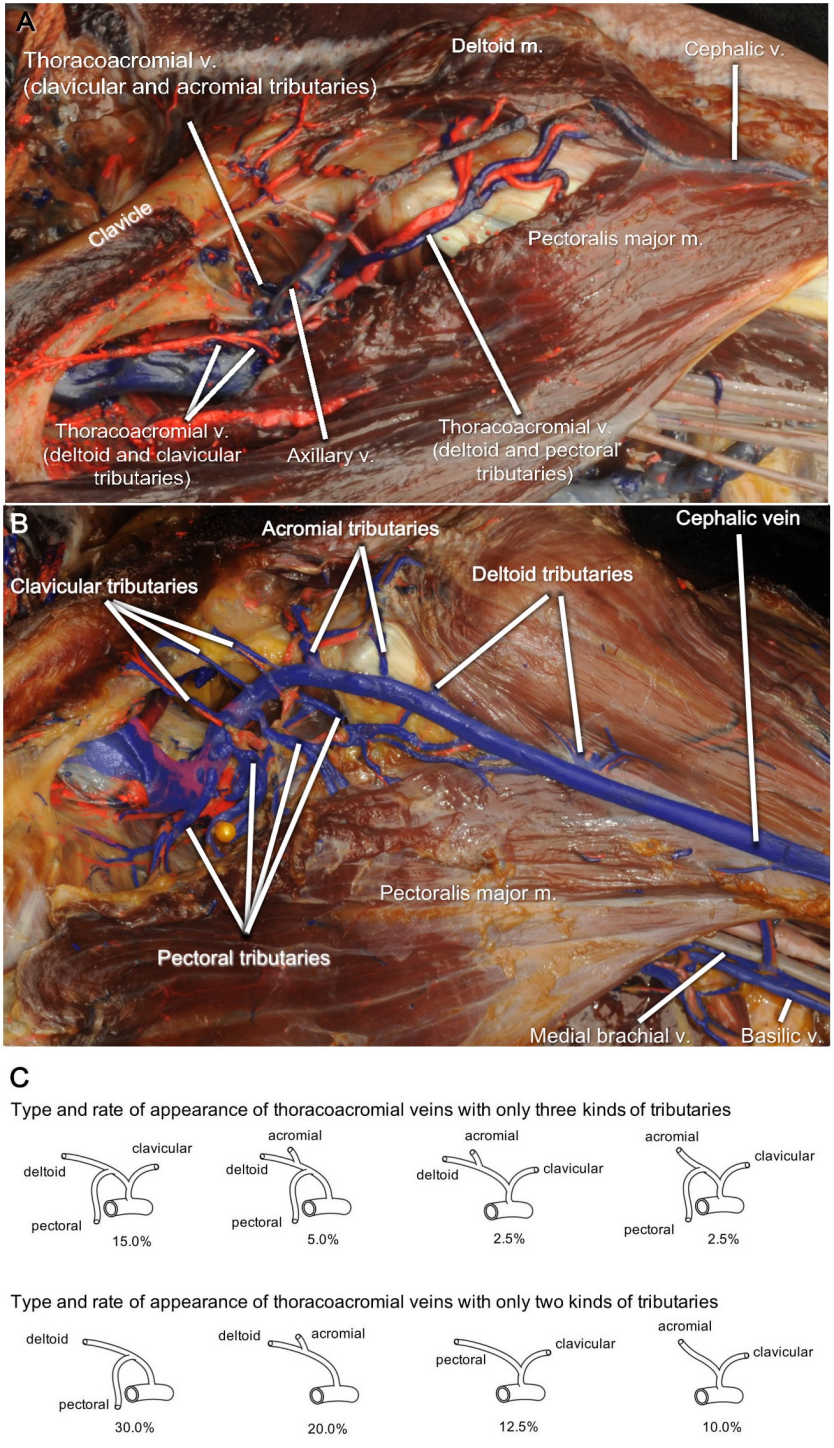
Thoracoacromial veins. **(**A) Three incomplete thoracoacromial veins are entering the cephalic vein in a left axilla. (B) Independent acromial, clavicular, deltoid, and pectoral veins drain into the cephalic vein in a left axilla. (C) The incomplete form of thoracoacromial veins receiving three or two kinds of venous tributaries. The frequencies of their existence in the upper limbs are shown. Note that there were upper limbs with multiple incomplete thoracoacromial veins.

It was found that many veins drained into the cephalic vein instead of axillary vein (Fig 1 and Table 1). First, the drainage of the thoracoacromial vein into the cephalic vein was observed in 70.0% of upper limbs (Fig 2A). In addition, acromial, pectoral, clavicular and deltoid venous tributaries, usually regarded as tributaries to the thoracoacromial vein, were observed to drain into the cephalic vein independently (Fig 2B). In 5.0% of upper limbs, the superior thoracic vein was also observed to drain into the cephalic vein. The indirect drainage of the anterior circumflex humeral vein, posterior circumflex humeral vein and deep brachial vein into the cephalic vein were also observed in this study.

**Table 1:** The destination of the veins.

Vein	to cephalic vein	to cephalic vein and axillary vein	to axillary vein	not existed	Total number of upper limbs
Thoracoacromial v.	25	3	5	7	40
Veins which did not drained into thoracoacromial vein					
Acromial v.	23	0	0	17	40
Clavicular v.	19	0	1	20	40
Deltoid v.	13	0	0	27	40
Pectoral v.	4	18	16	2	40
Superior thoracic v.	1	1	38	0	40
Lateral thoracic v.	0	0	38	2	40
Subscapular v.	0	0	32^a^	8	40
Anterior circumflex humeral v.	4 ^a^	0	36^a^	0	40
Posterior circumflex humeral v.	1 ^a^	2 ^a^	37^a^	0	40

^a^Including the veins which drained into the recipient vein indirectly via other veins

By contrast, the superior thoracic artery, thoracoacromial artery, lateral thoracic artery, subscapular artery, anterior circumflex humeral artery and posterior circumflex humeral artery which ramified from any artery other than the axillary artery was not observed in this study.

### The formation of the thoracoacromial vein and its drainage into the cephalic vein

The formation and drainage of the thoracoacromial vein did not mirror the origin and ramification of the thoracoacromial artery in nearly all of the specimens we dissected. A thoracoacromial vein which received the acromial, clavicular, deltoid, and pectoral tributaries and drained into the axillary vein was found in only one axillary specimen in the study. The other thoracoacromial veins had incomplete formation receiving only two or three of the acromial, clavicular, deltoid and pectoral tributaries. The most commonly observed type of incomplete thoracoacromial vein was one which received only the deltoid and pectoral tributaries (Fig 2C), and this type of thoracoacromial vein was observed in 30.0% of the upper limbs.

Sometimes, multiple incomplete thoracoacromial veins were observed in an upper limb. The number of incomplete thoracoacromial veins was one in 62.5% of upper limbs, two in 12.5% three in 5.0%.

Most of the incomplete thoracoacromial veins drained directly into the cephalic vein (62.5% of upper limbs). Others drained into the axillary vein or into both veins (Table 1).

### The independent acromial, clavicular, deltoid and pectoral veins

Unlike the arterial branches of the axillary artery, many of the acromial, clavicular, deltoid, and pectoral veins were not tributaries to the thoracoacromial vein, but instead drained into the cephalic vein independently (Fig 2B). The number of these independent veins in an upper limb ranged from 1–11. The average number of independent veins in an upper limb was 3.75, though this increased to 6.0 in those upper limbs without any thoracoacromial vein.

The most frequently observed independent vein was the pectoral vein which was identified in 95.0% of the upper limbs. It was followed by the acromial vein, the clavicular vein, and the deltoid vein which were observed in 57.5%, 50.0%, and 32.5% of upper limbs, respectively.

Many of the independent veins drained into the cephalic vein (Fig 2B and Table 1). Independent acromial vein and deltoid vein always drained into the cephalic vein. The drainage of the clavicular vein into the cephalic vein was more frequently observed than its drainage into the axillary vein with statistical significance (*p* = 0.00334). However, the pectoral vein did not have a tendency to drain into the cephalic vein.

### Drainage of the brachial veins

The lateral and medial brachial veins, the venae comitantes of the brachial artery, drained into the basilic vein or the axillary vein. They either ended separately, or merged to form the common brachial vein which joined the basilic vein or the axillary vein. In total, six different types of drainage of the lateral and medial brachial veins were observed (Table 2). The most common arrangement of the brachial veins was that the lateral and medial brachial veins joined the axillary vein separately without merging with each other.

**Table 2:** The formation of axillary vein by basilic and brachial veins.

Pattern of drainage		Number of upper limb
Lateral and medial brachial veins end separately		
	Both veins end in axilla and join the axillary vein	13
	One of the veins joins the axillary vein and the other joins the basilic vein	11
	Both veins end in arm and join the basilic vein	3
	Subtotal	27
Lateral and medial brachial veins merge before they end		
	Brachial veins merge in the axilla and join the axillary vein	2
	Brachial veins merge in the arm and join the axillary vein	6
	Brachial veins merge in arm and join the basilic vein	5
	Subtotal	13
Total		40

### The lateral venous channel

**Fig 3:**
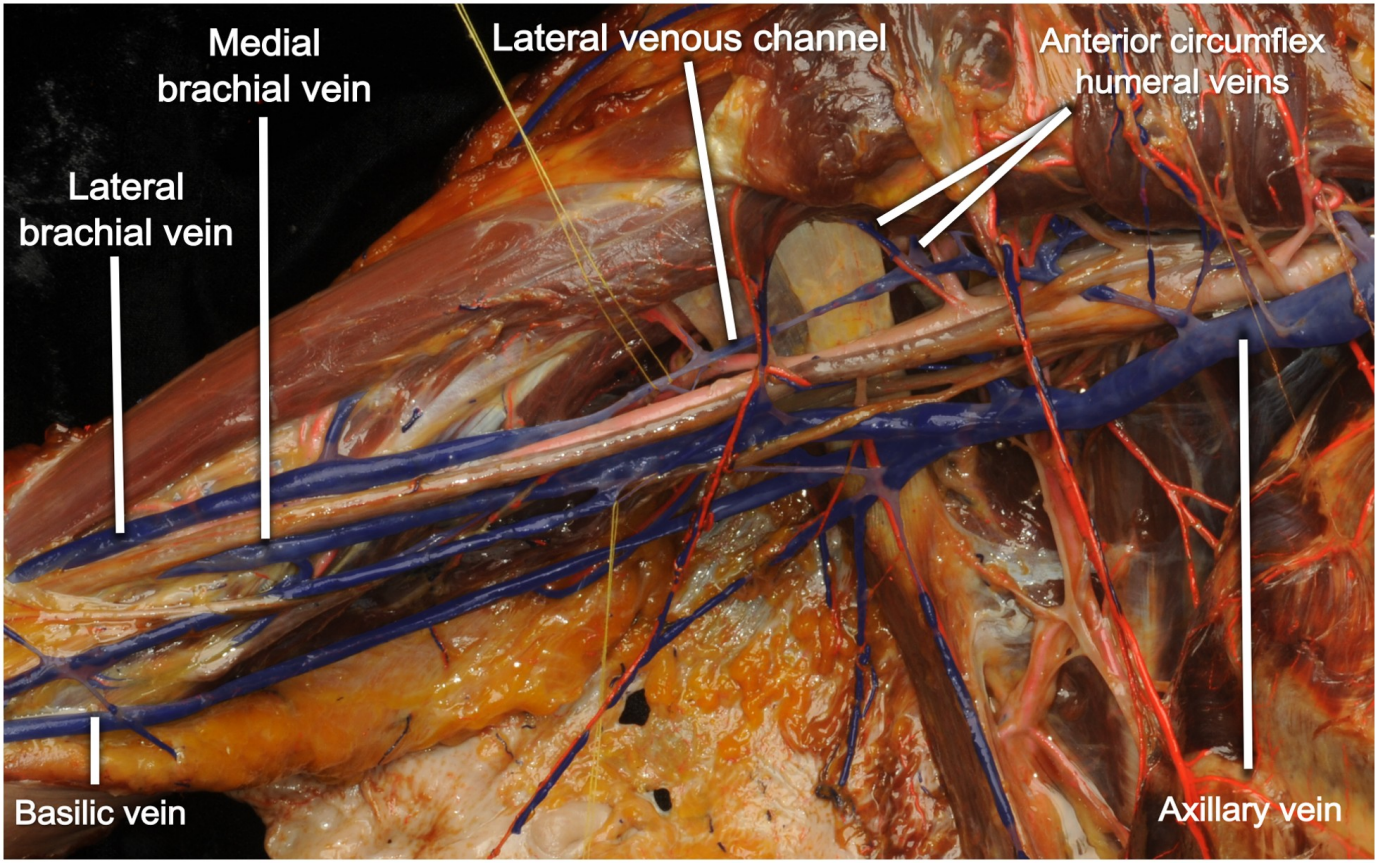
Lateral venous channel in a right axilla. A lateral venous channel is connecting the lateral brachial vein and the retropectoral part of the axillary vein. The anterior circumflex humeral veins are entering the lateral venous channel.

**Fig 4:**
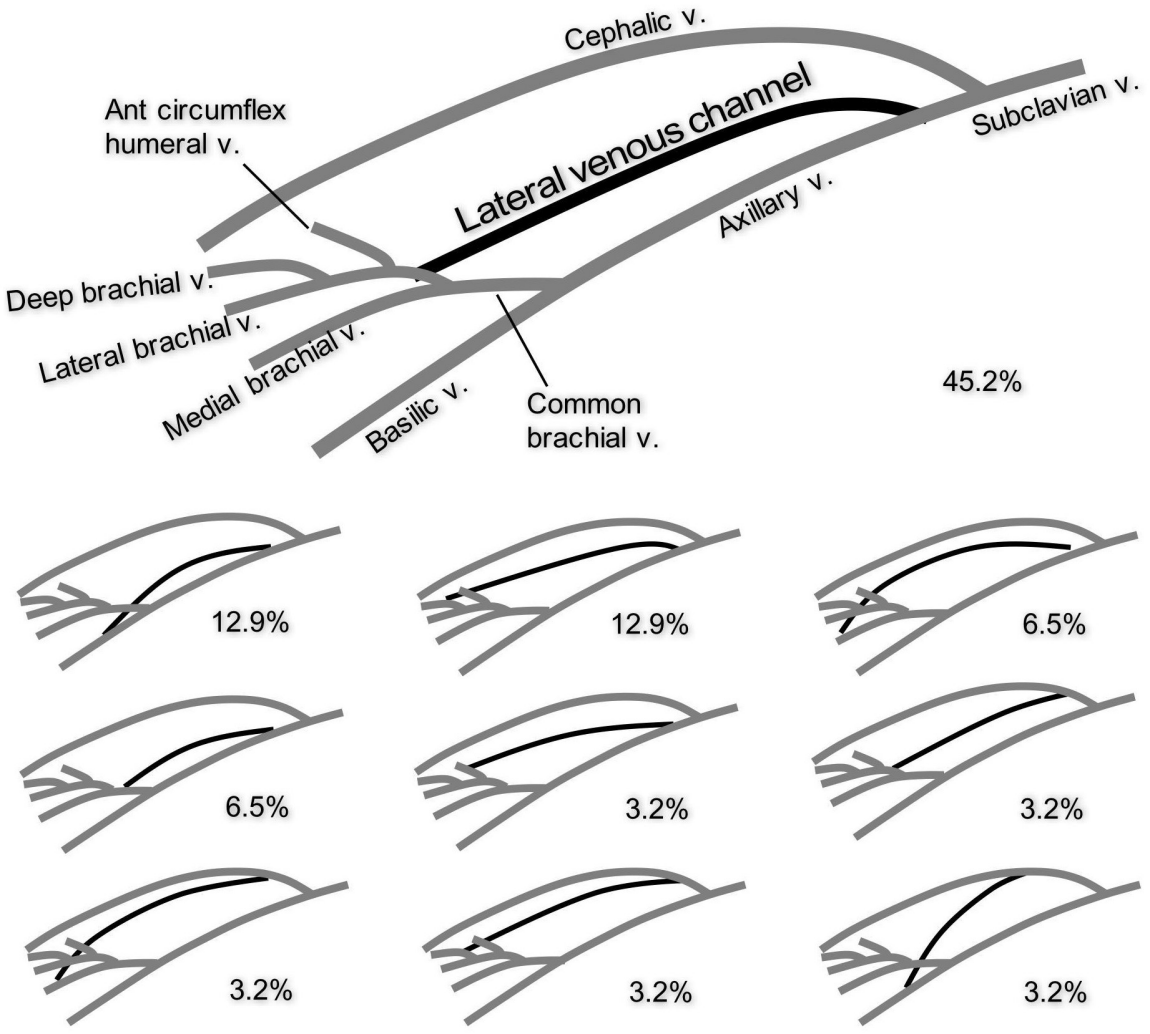
The distal and proximal connections of the lateral venous channel with the veins in the upper limb. The channel was distally connected to the basilic vein, common brachial vein, medial brachial vein, deep brachial vein and anterior circumflex humeral vein. The vein which was joined by the channel was the axillary vein or the cephalic vein. The most frequently observed connection was the connection between the lateral brachial vein and the axillary vein, which was observed in 45.2% of the upper limbs with the vein.

We observed the consistent presence of a venous channel along the posterolateral wall of the axilla, mainly superolateral to the axillary vein, axillary artery, and brachial plexus (Fig 3 and Table 3). The channels were identified in 77.5% of the upper limbs. They formed a venous connection between a vein in the distal part of the axilla and another vein in the proximal part (Fig 4). At its distal end, the venous channel had various connections to veins such as the lateral brachial vein (48.4% of the channels), the basilic vein (16.1%), the deep brachial vein (16.1%), the medial brachial vein (9.7%), the common brachial vein (6.5%), or the anterior circumflex humeral vein (3.2%). In contrast, the channel had more consistency with the connection of its proximal end, which was to either the axillary vein (87.1%) or the cephalic vein (12.9%). According to Fisher’s exact test, the lateral venous channel drained into the axillary vein more frequently than into the cephalic vein with a statistical significance (*p* = 0.00511).

**Table 3:** The course of lateral channel.

Course of lateral channel	Number of upper limb
Channels drained into axillary vein	27
lateral brachial vein ~ axillary vein	14
deep brachial vein ~ axillary vein	4
basilic vein ~ axillary vein	4
common brachial vein ~ axillary vein	2
medial brachial vein ~ axillary vein	2
anterior circumflex humeral vein ~ axillary vein	1
Channels drained into cephalic vein	4
lateral brachial vein ~ cephalic vein	1
deep brachial vein ~ cephalic vein	1
medial brachial vein ~ cephalic vein	1
basilic vein ~ cephalic vein	1
not existed	9
Total	40

The lateral venous channel was not just forming a bypass between distal and proximal parts of the axilla, but was also responsible for the drainage of veins in the axillary region such as the anterior circumflex humeral vein in 60.0% of the upper limbs, the posterior circumflex humeral vein (35.0%), or the deep brachial vein (27.5%).

Some of these veins eventually drained into the cephalic vein via the lateral venous channel. The anterior circumflex humeral vein drained into the cephalic vein via the lateral venous channel in 10.0% of the upper limbs. The posterior circumflex humeral vein drained into the cephalic vein via the lateral venous channel in 7.5%. The deep brachial vein drained into the cephalic vein via the lateral venous channel in 2.5%.

### The superior thoracic vein

Each specimen had single, double, or triple superior thoracic veins (average 1.33). Usually they drained into the axillary vein but the direct drainage of the superior thoracic vein into the cephalic vein was also observed in two upper limbs.

### The superficial vein of the axillary base

**Fig 5:**
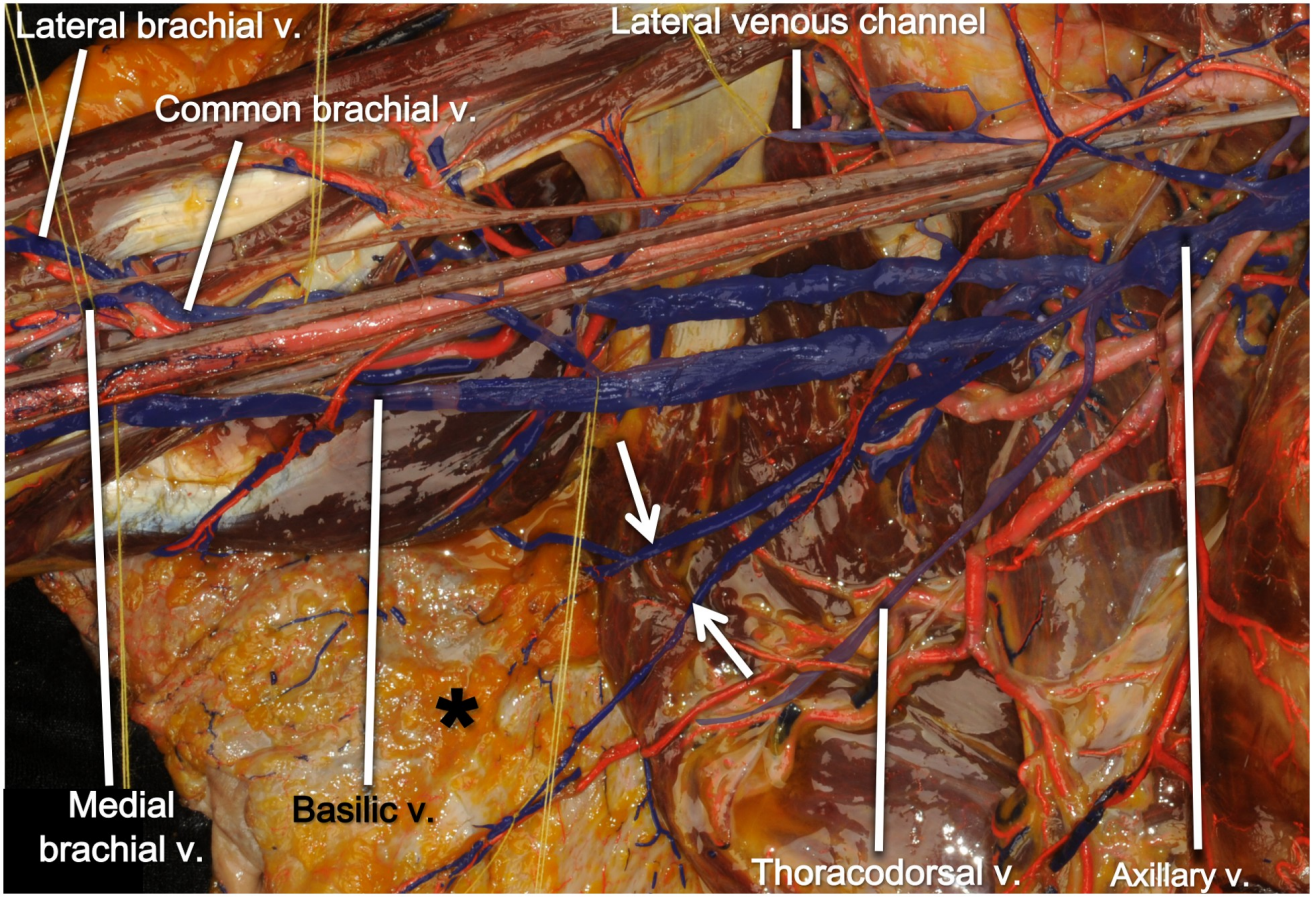
The superficial veins of the axillary base. Two veins (arrows) originate in the subcutaneous tissue of the base (asterisk) of a right axilla. They traverse the subcutaneous fat and end by draining into the infrapectoral part of the axillary vein.

Apart from the cephalic vein and the tributaries, there were large superficial veins which began in the subcutaneous tissue of the armpit, observed consistently in 95.0% of the upper limbs (Fig 5). The average number of these veins in a specimen was 2.08 (range: 1–5). They were situated in the subcutaneous fat of the axillary base and drained into the axillary vein in 92.5%. Its drainage into another superficial vein was rare. The drainage of the superficial veins was assisted by other vessels such as basilic, lateral brachial, medial brachial, common brachial, deep brachial, lateral thoracic and pectoral veins in 32.5% of the upper limbs.

### The subscapular vein and its tributaries

In contrast to the variable drainage pattern of the veins in the upper part of the axilla into the cephalic vein, the formation and drainage of the subscapular vein and its tributaries mirrored precisely the subscapular artery and its branches in nearly all of the specimens. The subscapular vein was observed in 80.0% of the upper limbs, and 96.9% of them drained into the axillary vein. In one upper limb, the subscapular vein drained into the medial brachial vein, which in turn drained into the third part of the axillary vein.

The subscapular vein did not exist in 20.0%. In the specimens without any subscapular vein, the circumflex scapular vein and the thoracodorsal vein drained into the axillary vein.

### The lateral thoracic vein

The lateral thoracic vein was present in 95.0% of upper limbs. The average number of lateral thoracic veins in an axilla was 1.68 (range: 1–3). In 80.0%, the lateral thoracic veins drained only into the axillary vein. In 12.5%, the lateral thoracic veins drained not only into the axillary vein, but also into the subscapular, thoracodorsal, or lateral brachial veins. In one upper limb, the lateral thoracic vein drained into the subscapular vein (Table 1).

### The anterior circumflex humeral vein and posterior circumflex humeral vein

Most of the anterior circumflex humeral vein and posterior circumflex humeral vein drained into the axillary vein or cephalic vein indirectly. The anterior circumflex humeral vein drained into axillary vein in 90.0% of the upper limbs which included indirect drainage via lateral brachial vein, deep brachial vein or lateral venous channel. The drainage into the cephalic vein was observed in 10.0% of upper limbs.

The posterior circumflex humeral vein drained into axillary vein in 92.5% of the upper limbs which included indirect drainage via lateral venous channel, circumflex scapular vein, subscapular vein, lateral brachial vein, anterior circumflex humeral vein, or deep brachial vein. In two upper limbs, the vein drained into both the axillary vein and cephalic vein. The drainage of posterior circumflex humeral vein only into the cephalic vein was observed in an upper limb, which was indirect.

### The deep brachial vein

The deep brachial vein was observed in 90.0% of the upper limbs. Although the deep brachial artery usually ramifies from the brachial artery distal to the axilla, the drainage of the deep brachial vein was consistently found in the axillary region. It drained mainly into the lateral venous channel (25.0%), lateral brachial vein (27.5%), axillary vein (10.0%), basilic vein (7.5%), medial brachial vein (7.5%), or common brachial vein (5.0%). The circumflex scapular, posterior circumflex humeral, and subscapular veins were also observed receiving the deep brachial vein.

### Difference in the ratio of venous drainage between axillary vein and cephalic vein and difference between body sides

According to Fisher’s exact tests on the drainage of each vein into axillary vein and cephalic vein, the thoracoacromial vein drained into the cephalic vein more frequently than into the axillary vein with statistical significance (*p* = 0.0127). In contrast, the superior thoracic vein, lateral thoracic vein, subscapular vein, anterior circumflex humeral vein, and posterior circumflex humeral vein drained into the axillary vein more frequently than into the cephalic vein with statistical significance (*p* < 0.001 for every test).

The pattern of drainage was not different between right and left upper limbs. According to Fisher’s exact tests, the destination of the veins did not have any statistically significant differences between left upper limb and right upper limb in this study (*p* > 0.05 in every test).

## Discussion

Extreme diversity of variation in the veins of the axillary region was discovered in this study. The cephalic vein has been often regarded simply as a cutaneous vein that originates in the dorsolateral side of the hand and collects the venous blood from the superficial structures of the lateral part of the upper extremity. Perhaps due to this, the cephalic vein is often sacrificed and widely used for various clinical purposes such as venous cut-down, creation of an arteriovenous fistula, carotid patching, and venous supercharging of congested areas [3, 22, 25, 26]. Our results suggest, however, that the venous drainage from a considerable part of the arm might also be charged into the cephalic vein. The cephalic vein is, according to the results of this study, a major recipient vessel for the thoracoacromial vein and its tributaries. The cephalic vein is also partly responsible for the drainage of the anterior circumflex humeral, posterior circumflex humeral, deep brachial and superior thoracic veins. These results imply that the cephalic vein is responsible for the venous drainage not only from the radial side of the hand and forearm but also from structures in the shoulder and upper lateral part of the arm.

The lack of homology in the course between of arteries and veins might be related with the difference in their development. The axillary artery and brachial artery are derived from the axial artery of the embryonic upper limb. On the other hand, the cephalic vein is derived from the preaxial vein and the axillary vein is derived from the embryonic postaxial vein which is not in a mirror image of embryonic axial artery. Unlike the arterial branches which have an axial artery to be connected to, the venous tributaries have the preaxial and postaxial veins [27–28]. According to our results, the thoracoacromial, acromial, clavicular, and deltoid veins drained to the cephalic vein more frequently, which means that they might be closely related with the preaxial vein during the development. By contrast, the other veins drained into the axillary vein more frequently, which means they might be closely related with the postaxial vein.

These underestimated but important roles of the cephalic vein might explain the complications of the cephalic venous access [2, 29]. The findings of this study suggest that many of the complications following invasive procedures might be related to the interruption of the venous drainage from the region around the shoulder to the cephalic vein. Any invasive procedures on the cephalic vein would affect the drainage of the thoracoacromial vein and tributaries in many cases, as well as the drainage of circumflex humeral veins, deep brachial vein and superior thoracic vein in rare instances.

In addition to the drainage of the thoracoacromial vein into the cephalic vein, the direct independent drainage of the deltoid, clavicular, acromial and pectoral veins into the cephalic vein has also been unrecognized. However, knowledge of their course may useful in planning new kinds of venous approach. In a feasibility study investigating the implantation of an intravenous port through the deltoid tributary of the thoracoacromial vein, immediate catheter implantation via the tributary was possible in only 47.4% of the patients, as the vessel was found to be too tortuous for the procedure in the remaining patients [24]. One possible explanation for the difficulties might be that the tortuous vessels of the deltoid venous tributaries they encountered during the procedures were not heading to the axillary vein, but were instead tributaries to the cephalic vein, like many of those found in this study.

Another finding which has not been described before was the existence of a superficial vein of the axillary base under the skin of armpit. As the exploration for a safe alternative entry vessel is crucial for the treatment of patients without other feasible veins [24], the discovery of the consistent presence of this superficial vein of the axillary base might be beneficial, especially as the vein is positioned close to the axillary and subclavian veins. In contrast to the axillary vein which usually requires ultrasound guidance for its identification, this superficial vein is situated in the subcutaneous tissue and is visible under the skin. Another advantage of this superficial vein is that the armpit is a region that is often protected from traumatic injury, accident or burn which can damage other superficial veins in the upper limb. Therefore, it might be used for venous access, especially when other superficial veins are impossible.

The consistent finding of the lateral venous channel is also interesting. The lateral venous channel seemed to be primarily responsible for the drainage of the anterior circumflex humeral vein and partly responsible for the drainage of the posterior circumflex humeral vein and the deep brachial vein. In addition to these roles in venous drainage, it may also function as a venous bypass route in the event of compression of the axillary vein, as this channel connects the distal and proximal regions of the axilla.

The presence of the lateral venous channel, the drainage of the thoracoacromial venous tributaries into the cephalic vein, the drainage of the deep brachial vein into the axillary vein, and the discovery of the cutaneous vein of the armpit (superficial vein of the axillary base) indicates that the venous system in the axilla does not normally mirror the branches of the axillary artery and that the venous anatomy of the axilla should be distinguished from the arterial system.

The limitation of this study was that the cadaveric veins without any blood pressure would go flat and less elastic so that the measurement of their size was not reliable enough to report.

## Conclusions

The anatomy of the axillary vein and its tributaries did not mirror the axillary artery and its branches. First, the veins in the axillary area drained not only into the axillary vein but also into the cephalic vein. Especially, the thoracoacromial vein and the tributaries drained into the cephalic vein more frequently. In addition, the lateral venous channel was partly responsible for the drainage from posterolateral part of arm. The superficial vein of the axillary base is not connected to the cephalic vein or the basilic vein, but drained directly into the axillary vein, and could potentially be used for intravenous access.
