# Correction: The axillary vein and its tributaries are not in the mirror image of the axillary artery and its branches

**DOI:** 10.1371/journal.pone.0230471

**Published:** 2020-03-10

**Authors:** HyeYeon Lee, JongHo Bang, SooJung Kim, HeeJun Yang

All four authors should not have been attributed equal contribution to this work.
